# PI3K Isoform-Specific Regulation of Leader and Follower Cell Function for Collective Migration and Proliferation in Response to Injury

**DOI:** 10.3390/cells11213515

**Published:** 2022-11-07

**Authors:** Morgan D. Basta, A. Sue Menko, Janice L. Walker

**Affiliations:** 1 Department of Pathology, Anatomy and Cell Biology, Sidney Kimmel Medical College, Thomas Jefferson University, Philadelphia, PA 19107, USA; 2 Department of Ophthalmology, Sidney Kimmel Medical College, Thomas Jefferson University, Philadelphia, PA 19107, USA

**Keywords:** collective migration, leader cell, follower cell, wound healing, PI3K, p110α

## Abstract

To ensure proper wound healing it is important to elucidate the signaling cues that coordinate leader and follower cell behavior to promote collective migration and proliferation for wound healing in response to injury. Using an ex vivo post-cataract surgery wound healing model we investigated the role of class I phosphatidylinositol-3-kinase (PI3K) isoforms in this process. Our findings revealed a specific role for p110α signaling independent of Akt for promoting the collective migration and proliferation of the epithelium for wound closure. In addition, we found an important role for p110α signaling in orchestrating proper polarized cytoskeletal organization within both leader and wounded epithelial follower cells to coordinate their function for wound healing. p110α was necessary to signal the formation and persistence of vimentin rich-lamellipodia extensions by leader cells and the reorganization of actomyosin into stress fibers along the basal domains of the wounded lens epithelial follower cells for movement. Together, our study reveals a critical role for p110α in the collective migration of an epithelium in response to wounding.

## 1. Introduction

Collective migration involves the coordinated movement of cells that maintain contact with one another as they migrate [1,2,3]. It is an essential aspect of morphogenesis during embryonic development, tissue regeneration, and wound healing, as well as cancer progression [1,2,3]. During collective migration, leader cells and follower cells must establish front-to rear polarity and synchronize their function to undergo directional movement [2,4,5]. To this end, leader and follower cells need to coordinate their cytoskeletal activity and mechanically couple to maintain contact with one another and produce traction forces to drive the group of cells forward. An important feature of leader cells is their ability to polarize and form protrusions to drive movement [1,2,5]. Less understood is how follower cells actively participate in collective migration. One way is through their formation of cryptic lamellipodia protrusions along their basal domains to help produce traction forces to promote migration [2,6,7,8]. Additionally, critical are cell–cell junctions, the fundamental way that biomechanical inputs are communicated to the group of moving cells [9]. A balance of myosin II generated forces are needed to maintain connectivity between cells and provide forces to propel the group of cells forward [7,10,11]. Together, leader and follower cells respond to cues such as chemokine signals for polarized directional movement. However, much remains to be discovered regarding which signaling cues coordinate leader and follower cell behavior and orchestrate cooperation of their cytoskeletal activities for collective migration in response to injury. 

Candidate signaling cues for regulating leader and follower cell behavior in response to injury include the class I phosphatidylinositol-3-kinases (PI3Ks) due to their ability to impact cells at multiple levels including sensing chemotactic gradients, generating protrusion formation, regulating cell-matrix and cadherin mediated cell–cell junction formation and promoting proliferation [12,13,14,15,16,17,18]. Furthermore, class I PI3Ks can exist in multiple isoforms that can sense diverse signaling inputs, adding another level of regulation [19]. PI3K consists of a regulatory (i.e., p85) and 110 kDa (p110) catalytic subunit [19,20,21]. Its catalytic subunit can be subdivided into subclass IA isoforms consisting of p110α, p110β, p110δ and subclass IB that consists of the isoform p110γ [19,20,21]. While p110α, p110β are ubiquitously expressed p110γ and p110δ exhibit more restrictive expression patterns [19,20,21]. PI3K signaling occurs at the plasma membrane to generate the lipid product phosphatidylinositol-3,4,5 trisphosphate (PIP3) by the phosphorylation of the 3′ hydroxyl group on the inositol head group of phosphatidylinositol-4,5-bisphosphate (PIP2) [19]. PIP3 can then activate downstream substrates including Akt and Rac GEFs such as Vav and PREX [18,22]. Moreover, PI3Ks can crosstalk with Rho GTPase family members, which are connected to regulating the cytoskeleton and cadherin cell–cell junctions, essential aspects of collective migration [23]. Compared to single cell migration far fewer studies exist regarding the role of PI3K in collective migration. In a study using Madin-Darby canine kidney (MDCK) cells, PI3K signaling was restricted to leader cells and critical to leader cell survival and proper collective migration [24]. Even less is understood about the role of PI3K isoforms in coordinating leader-follower cell behavior for collective migration in response to injury. 

We previously established an ex vivo post-cataract surgery model to investigate leader-follower cell behavior in response to injury. In this model it is possible to study leader cell directed collective movement of wounded lens epithelial cells across a native basement extracellular matrix (ECM) environment (the cell-denuded lens capsule) for wound healing [7,25]. Wound healing across the lens basement membrane is completed by day 3 post-injury [7,25,26]. Here, collective migration involves heterotypic cell interactions between CD44+ resident immune leader cells and the follower wounded lens epithelia cells [27]. In response to injury, resident immune cells become activated to migrate to the leading edge to serve as leader cells to direct the movement of the follower cells, the wounded lens epithelial cells [27]. CD44+ resident immune leader cells extend vimentin-rich protrusions at the wound edge and vimentin function is required for extension and wound healing [26,28]. The collectivity of the wounded lens epithelial cells is maintained by apical ZO-1 junctions as well as N-cadherin junctions that extend all along their apical-lateral cell–cell membranes [7]. Active myosin forces are found at apical cell–cell junctions to maintain interconnection of the wounded epithelial cells [7]. Basally, wounded lens epithelial follower cells reorganize actin from a cortical distribution into stress fibers that connect cryptic lamellipodia containing atypical cadherin junctions between cells along the basal substrate [7]. These cryptic lamellipodia are enriched for active myosin II at the front of the cell abutting the tailing edge of the cell behind it in the moving monolayer [7]. Apical and basal myosin function was critical to proper organization of the wounded epithelial monolayer and wound healing as treatment with blebbistatin resulted in loss of proper apical-basal polarity, changes in cell shape and an uncontrolled migration response leading to faster wound healing [7]. In the current study we used the ex vivo post-cataract surgery model to investigate PI3K class I specific isoform-specific function in orchestrating proliferation, leader and follower cell cytoskeletal organization, myosin II activity, and cell behavior in response to wounding. Our findings indicate that resident immune leader cell and wounded epithelial follower cell function are regulated in a PI3K isoform specific manner for wound healing. 

## 2. Materials and Methods

### 2.1. Model and Treatments

Ex vivo post-cataract surgery cultures were prepared on isolated lenses from E15 chick embryos (Poultry Futures, Lititz, PA, USA) using methods previously described [7,26,29,30]. Briefly, a method paralleling cataract surgery is performed on isolated lenses to remove the lens fiber cells leaving behind epithelial cells that are tightly adherent to the lens capsule. Star shaped explants are created in which wound healing can be followed across the fiber-cell denuded basement membrane lens capsule. Explants were cultured in Media 199 (Invitrogen, Calsbad, CA, USA) with 1% penicillin/streptomycin (Mediatech-Cellgro, Manassas, VA, USA), 1% L-glutamine (Mediatech-Cellgro), and 10% fetal calf serum (Invitrogen). For wound healing studies, ex vivo post-cataract surgery cultures were treated at either time 0 or Day 1 for 24hr or until Day 3. To inhibit PI3K p110 isoforms, 1 μM HS-173 (Selleckchem, Houston, TX, USA), 3 μM GSK2636771 (Selleckchem, Houston, TX, USA), and 3 μM Duvelisib (Selleckchem, Houston, TX, USA) were used. To inhibit AKT, 3 μM MK-2206 (Selleckchem, Houston, TX, USA) was used. Cultures were fixed in 4% formaldehyde or extracted in Triton X-100/Octyl glucoside (Triton/OG) extraction buffer (44.4 mM n-octyl β-d-glucopyranoside, 1% Triton X-100, 100 mM NaCl, 1 mM MgCl_2_, 5 mM EDTA, 10 mM imidazole) with protease/phosphatase inhibitor cocktail (Cell Signaling Technology, Aldrich, Danvers, MA, USA). 

### 2.2. Phase Microscopy and Wound Healing 

Phase contrast images at the leading edge of the wound were taken of Day 1 or Day 2 ex vivo post-cataract surgery cultures with a Nikon Eclipse T*i* microscope. Images of wound healing were taken daily (Day 0, Day 1, Day 2, Day 3) using an AZ100 Nikon microscope and wound areas were measured using NIS elements image-analysis software. 

### 2.3. Immunofluorescence, EdU-Labeling, and Confocal Microscopy

For immunofluorescence staining, fixed cultures were permeabilized with 0.25% Triton-X-100, blocked with 5% goat serum prior to incubation with primary antibodies. Primary antibodies for immunolabeling included: Vimentin (AMF-17b, DSHB, Iowa City, IA, USA) Phospho-Myosin Light Chain 2 (Thr18/Ser19, Cell Signaling 3674, Danvers, MA, USA). The AMF-17b monoclonal antibody to vimentin was deposited by Fulton, A.B. Cultures were counterstained with DAPI (Biolegend, San Diego, CA, USA) to identify nuclei and fluorescent-conjugated Phalloidin (Invitrogen, Waltham, MA, USA) to label filamentous actin. Following primary antibodies, cultures were incubated with fluorescent conjugated secondary antibodies (Jackson Immunoresearch, West Grove, PA, USA). To detect proliferating cells, cultures were EdU-labeled with 10 μM EdU for 30 min and the Click-iT EdU imaging kit (Invitrogen, Carlsbad, CA, USA) was used following manufacturer’s instructions. Images were taken on a confocal Zeiss 800. Z-stacks were collected at 0.33 μm and 3D-structural images were created from Zeiss 800 confocal microscope using Imaris ×64 v9.5.1 software surface rendering tool.

### 2.4. Western Blot and Wes Protein Analysis

Ex vivo post-cataract surgery cultures were extracted on Time 0. For western blot analysis of PI3K p110 isoform expression, 10–20 μg of protein was loaded per lane and separated by 4–12% SDS-PAGE and transferred at 4° on PVDF membranes. Membranes were blocked using 5% milk for 1 h and incubated overnight at 4° with primary antibodies: PI3K p110α (4249, Cell Signaling, Danvers, MA, USA), p110β (sc-376641, Santa Cruz, Dallas, TX, USA), and p110γ (sc-7177, Santa Cruz, Dallas, TX, USA). Following primary antibody incubation, HRP-conjugated mouse, or rabbit secondary antibodies (Bio-Rad, Hercules, CA, USA) were incubated for 1hr, and membranes were exposed to ECL+ chemiluminescence-substrate-detection reagent using ProteinSimple machine. For Wes protein analysis ex vivo post-cataract surgery cultures were extracted on day 3 and 1 μg of protein was loaded per well. For analysis of pAKT and total AKT, phosphor-AKT (4060, Ser473, Cell Signaling, Danvers, MA, USA) and AKT antibody (9272, Cell Signaling, Danvers, MA, USA) were added and the protocol was followed per manufacturer’s instructions using Simple Western Automated Western Blot Wes Instrument. (Biotechne, Minneapolis, MN, USA) as previously described [31].

### 2.5. Statistical Analysis

Statistical analyses for data quantification were performed using GraphPad Prism Software (GraphPad, San Diego, CA, USA). Data is presented at ±SEM from 2 or 3 independent experiments and appropriate statistical analyses were performed for all quantified experiments according to the figure legends.

## 3. Results

### 3.1. Collective Migration for Wound Healing in Response to Cataract Surgery Injury Occurs in a PI3K Isoform-Specific Manner Independent of Akt Signaling

**Figure 1:**
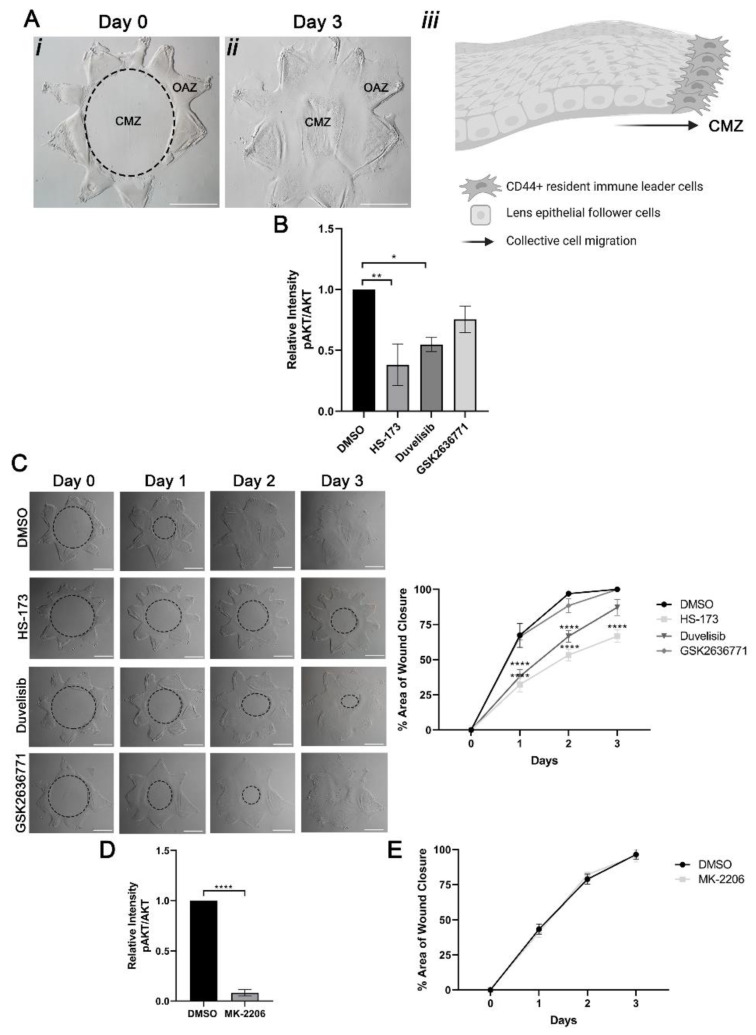
Wound healing in response to cataract surgery injury occurs in a PI3K isoform-specific manner. (**A**) Depiction of ex vivo post-cataract surgery culture to study mechanisms of wound repair across the cell-denuded endogenous basement membrane in the center of the lens capsule. Ex vivo post-cataract surgery explant on day 0 depicts the wounded lens epithelial cells within the original attachment zone (OAZ) and the cell-denuded central migration zone (CMZ) (i). In response to injury, collective migration across the endogenous basement membrane in the CMZ is driven by leader cells that direct the migration of the wounded epithelial follower cells (ii), arrow depicts the direction of wound healing. Collective migration across the CMZ for wound repair is completed by day 3 post-injury (iii). (**B**,**C**) Ex vivo post-cataract surgery explants were treated from Time 0 through Day 3 with individual PI3K isoform inhibitors, HS-173 for p110α; GSK2636771 for p110β; or Duvelisib for p110δ/γ. (**B**) Graph from Wes analysis depicts the efficacy of individual PI3K isoform inhibitors compared to vehicle control by examining the relative intensity of the PI3K effector, phosphorylated-AKT (pAKT) relative to total AKT expression compared to GAPDH (loading control). (**C**) Phase microscopy shows open wound area (dotted black circle) for vehicle vs. PI3K inhibitor treatment from Day 0 through Day 3. Graph depicts % area of wound closure. Results show that blocking p110α with HS-173 had the largest impact on inhibiting wound closure. (**D**,**E**) Ex vivo post-cataract surgery explants were treated with vehicle (DMSO) or the AKT specific inhibitor, MK-2206 from Time 0 through Day 3. (**D**) MK-2206 suppressed pAKT relative to total AKT expression and GAPDH (loading control). (**E**) Graph of % wound closure shows that treatment with MK-2206 had no effect on wound closure. *p*-values were determined from one-way ANOVA (**B**), two-way ANOVA with multiple comparisons (**B**,**C**) or unpaired *t*-test (**D**,**E**). (**B**, * *p* < 0.05, ** *p* < 0.01; **C**, **** *p* < 0.0001 and **D** **** *p* < 0.0001). Data is expressed as ± SEM from 2 (**D**,**E**) or 3 (**B**,**C**) independent experiments. Magnification bars = 1000 μm (**A**,**C**).

Using our previously established ex vivo post-cataract surgery chicken wound healing explant cultures (Figure 1A) we investigated the role of class I PI3K isoforms in signaling collective migration in response to lens injury. To create these wounded explant cultures, a cataract surgery microsurgery is performed on isolated lenses, which removes the differentiated fiber cells that comprise the bulk of this tissue. This process leaves behind the lens epithelial cells that are tightly adherent to the basement membrane capsule along with the associated lens resident immune cells. Additional cuts are made to the lens capsule to flatten the explant on the culture dish, creating a star-shaped culture in which the lens epithelial and resident immune cells are located cell-side up in the points of the star. This region is termed the original attachment zone (OAZ) (Figure 1Ai). In response to injury, resident immune cell leader cells rapidly populate the wound edge and direct the wounded lens epithelial follower cells to migrate collectively across the cell denuded basement membrane to close the wound (Figure 1Aii, see model). This region is referred to as the central migration zone (CMZ). Wound closure is typically completed by D3 post-injury (Figure 1Aiii).

A previous study showed that class I PI3K isoforms p110α, p110β and p110γ are all expressed in the lens [32]. Consistent with these findings we observed the expression of p110α, p110β and p110γ class I PI3K p110 isoforms in the wounded explant cultures at time 0 (Supplemental Figure S1). To determine the potential function of these three class I PI3K p110 isoforms in wound closure, ex vivo post-cataract surgery cultures were treated with vehicle or PI3K p110 isoform specific inhibitors, HS-173 (HS) to p110α; GSK2636771 (GSK) to p110β, or Duvelisib (Duv) to p110δ/γ, from time 0 through day 3. In DMSO-treated cultures the wound was closed within three days (Figure 1C, DMSO, D3). Impact of each p110-specific inhibitor on activation of AKT (pser-473 AKT/total AKT), a principal PI3K downstream effector, was determined on day 3 post-treatment (Figure 1B). The greatest level of inhibition of AKT signaling was observed with the p110α inhibitor HS-173 in the wounded lens. Next most impactful inhibition of AKT activation occurred with the p110γ/δ inhibitor Duvelisib, with the p110β inhibitor GSK2636771 having the least impact (Figure 1B). These findings show that the link of these different p110 isoforms to AKT downstream signaling was isoform specific in the lens. Similarly, suppression of PI3K p110α signaling had the greatest impact on wound closure, with only approximately 67% closure on day 3 post-injury (Figure 1C). In contrast, treatment with GSK2636771 to block p110β had no effect on wound closure and showed a similar wound healing trend as vehicle control (Figure 1C). Treatment with Duvelisib to block p110γ/δ also slowed wound repair, but not as pronounced as inhibition of p110α, with 87% closure by D3 (Figure 1C). These studies suggest that both p110α and p110γ/δ function in promoting wound healing in response to lens injury.

There are many downstream signaling effectors of PI3K, most common among them AKT and Rac. We examined whether the impact of PI3K signaling on promoting lens wound repair involved signaling through AKT. For these studies, ex vivo wounded explants were treated with vehicle DMSO or the AKT specific inhibitor MK-2206 (MK) from time 0 through day 3 (Figure 1D,E). Treatment with MK effectively suppressed activation of AKT (pAKT/total AKT) expression (Figure 1D). However, blocking Akt had no effect on closure of the cataract surgery wound (Figure 1E). In the presence of both vehicle and MK treatment, wound closure was complete by day 3 post-injury (Figure 1E). These findings show that PI3K isoform-dependent wound closure involves an alternative downstream effector(s).

### 3.2. p110α Is Required to Promote and Maintain Extension of Vimentin-Rich Lamellipodial Protrusions by Leader Cells

**Figure 2:**
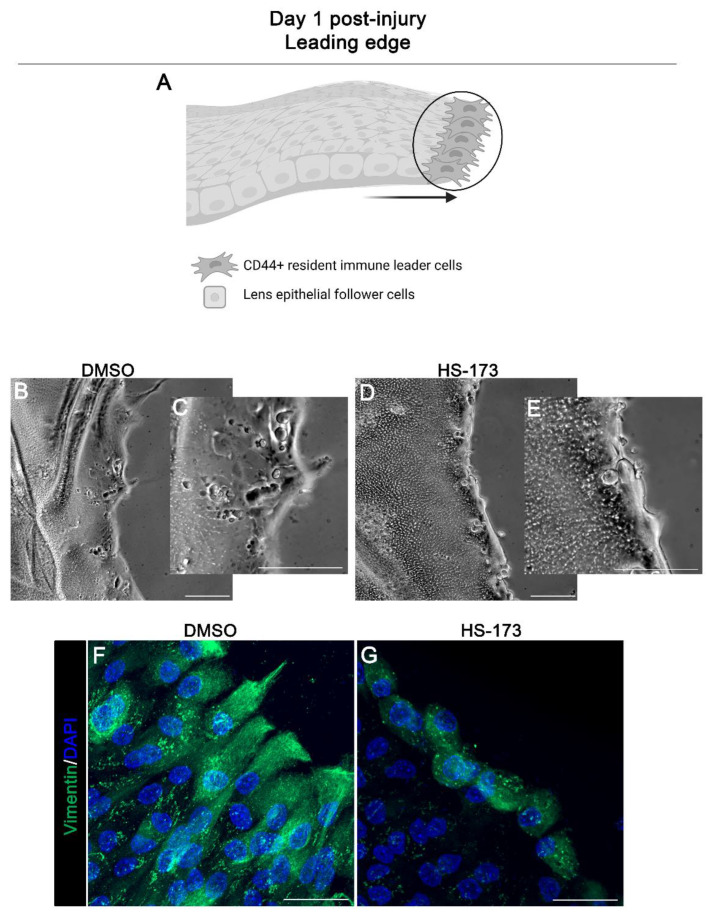
p110α is required to initiate extension of vimentin rich lamellipodial of leader cells. (**A**) Model of CMZ highlighting our region of interest (leader cells, black circle) for these studies. (**B**–**G**) Ex vivo post-cataract surgery explants were treated with HS-173 to inhibit p110α from Time 0 for 24 h (Day 1 post-injury). (**B**) Phase contrast imaging shows that in the presence of DMSO leader cells extend protrusions at the wound edge. (**D**) In contrast, HS-173 blocked leader cell protrusion at the wound edge. Regions in (**B**,**D**) are shown at higher magnification (**C**,**E**). (**F**,**G**) Cataract surgery explants day 1 post-treatment were immunolabeled for vimentin (green) and counterstained for DAPI (blue). Confocal images revealed vimentin-rich lamellipodia extensions at the leading edge were blocked by HS-173 treatment (**G**) compared to vehicle control (**F**). Magnification bars = 50 μm (**B**–**E**) and 20 μm (**F**,**G**).

To examine the mechanism of how p110α promotes collective migration following wounding we determined the specific effects of blocking p110α on the function of resident immune cells at the leading edge of the cataract surgery wound. Our previous studies showed that the leader cells, a vimentin-rich cell population, play an essential role in mediating lens post-cataract surgery wound healing, and that vimentin function is required for their extension of lamellipodial processes along the basement membrane substrate [26]. Here, we investigated the impact of blocking p110α function on vimentin-rich lamellipodial extension by leader cells (modeled in Figure 2A). Ex vivo post-cataract surgery cultures were exposed to the HS-173 inhibitor to block p110α from day 0 through day 1 post-wounding and compared to vehicle DMSO treated controls. The extension of processes by leader cells was examined by both phase microscopy imaging (Figure 2B–E) and by confocal microscopy imaging after immunolabeling for vimentin (Figure 2F,G). Phase contrast images show that the HS-173 p110α inhibitor blocked extension of normal protrusions by leader cells at the wound edge on day 1 (Figure 2B–E). Immunolabeling for the cytoskeletal protein vimentin highlights this failure to extend lamellipodial processes along the substrate (Figure 2G, HS-173) that are normally rich in vimentin (Figure 2F, DMSO). In the presence of HS-173 the leader cells at the at the wound edge remained round (Figure 2G).

**Figure 3:**
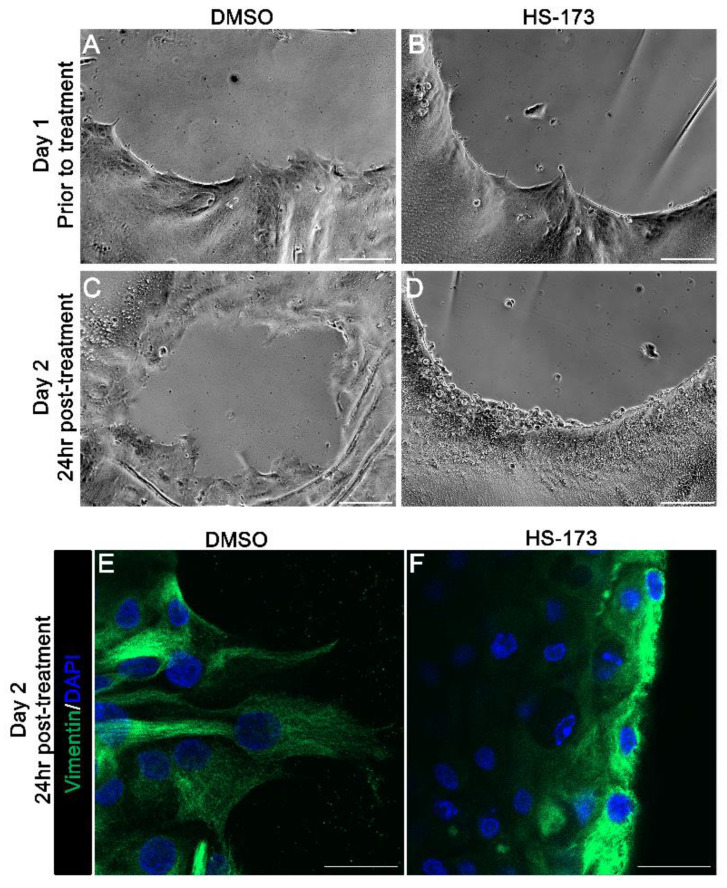
p110α is required to maintain the extension of vimentin-rich lamellipodia of leader cells. (**A**–**F**) Ex vivo post-cataract surgery explants were treated with vehicle (DMSO) or HS-173 to inhibit p110α from Day 1 for 24 h (Day 2 post-injury). (**A**,**B**) Phase contrast imaging on Day 1 shows the extension of protrusions by leader cells at the wound edge prior to treatment. (**C**,**D**) In contrast, phase contrast imaging on Day 2 (24 h post-treatment) revealed that HS-173-treated leader cells failed to maintain protrusions compared to vehicle controls. (**E**,**F**) Day 2 explants following 24 h of treatment were immunolabeled for vimentin (green) and counterstained for DAPI (blue). Confocal images revealed that maintenance of vimentin-rich lamellipodia extensions was blocked by HS-173 treatment (**F**) compared to vehicle controls (**E**). Magnification bars = 50 μm (**A**–**D**) and 20 μm (**E**,**F**).

**Figure 4:**
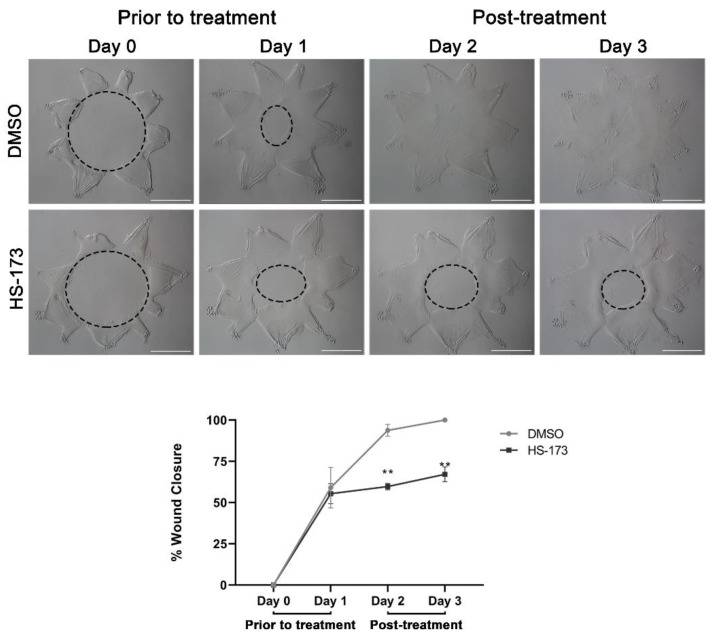
p110α is required throughout the wound healing process to sustain wound closure. Ex vivo post-cataract surgery explants were treated with HS-173 to inhibit p110α from Day 1 to Day 3 post-injury. Phase microscopy and wound area measurements show the wound area prior to treatment on Day 0 and Day 1 and post-treatment with vehicle or HS-173 on Day 2 and Day 3 (dotted black circle). Graph depicts % wound closure and revealed that HS-173 is required throughout the wound healing process to drive wound closure. *p*-values were determined from two-way ANOVA with multiple comparisons (** *p* < 0.01). Data is expressed as ±SEM from 3 independent experiments. Magnification bars = 1000 μm.

The extension of lamellipodial processes by leader cells at the wound edge is required for directional persistence. Our findings showed that p110α is necessary to initiate extension of these vimentin rich protrusions. Next, we asked whether p110α is also required to maintain lamellipodial processes that had been already extended by leader cells along the substrate. For these studies, the HS-173 p110α inhibitor was added to the cataract surgery explant cultures at day 1 post-injury, after the extension of the vimentin-rich lamellipodial protrusions by leader cells. Phase images were acquired at the wound edge both prior to exposure to the inhibitor (Figure 3A,B, D1 post-injury) and following 1 day exposure to HS-173 (Figure 3D, D2 post-injury) or DMSO vehicle control (Figure 3C, D2 post-injury). In vehicle controls, the leader cells continued to extend protrusions along the cell-denuded basement membrane substrate in D2 wounded explants (Figure 3C). Exposure to the HS-173 inhibitor resulted in failure of leader cells to maintain these cellular protrusions (Figure 3D). Labeling for vimentin further confirmed these findings showing that p110α is required to maintain the extension of vimentin-rich lamellipodial protrusions by leader cells (Figure 3F). Treatment with HS-173 caused the collapse of vimentin-rich extensions (Figure 3F) that are commonly observed in the control explants (Figure 3E). Similar to blocking p110α from time 0, exposure of the cataract surgery wounded cultures to the HS-173 inhibitor from D1 through D3 also impaired wound closure, with the wound still remaining 33% open at culture D3 (Figure 4). These studies show that p110α function is critical to regulate both the formation and maintenance of vimentin rich lamellipodial extension by leader cells and suggest p110α function is required throughout the wound healing process for wound closure.

### 3.3. p110α Regulates the Reorganization of F-Actin along the Basal Surface of the Wounded Epithelial Cells

**Figure 5:**
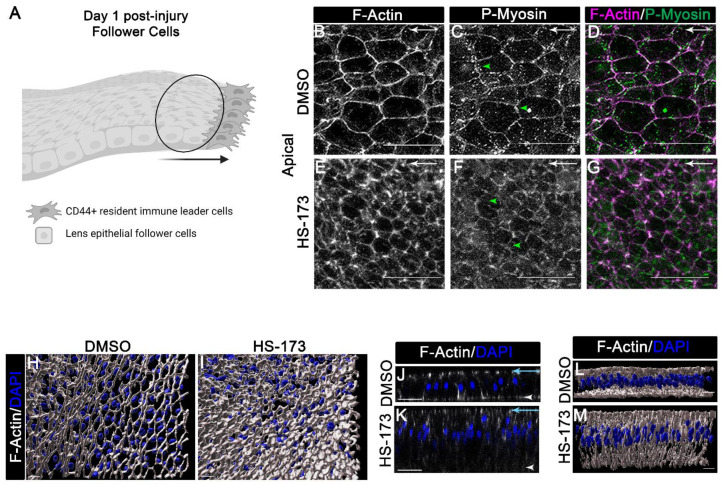
Impact of blocking p110α on the apical organization of the wounded lens epithelial follower cells post-injury. (**A**) Model of CMZ highlighting our region of interest (follower cells, black circle) for these studies. (**B**–**M**) Ex vivo post-cataract surgery explants were treated with vehicle control or HS-173 to inhibit p110α from Time 0 for 24 h (Day 1 post-injury). Cultures were labeled with phalloidin to identify filamentous actin (F-actin, white (**B**,**E**), purple (**D**,**G**)) and immunolabeled for active myosin (p-myosin, white (**C**,**F**), green (**D**,**G**)). (**B**–**G**) Confocal images of follower cells focused apically revealed a cortical distribution of actomyosin at cell–cell border with vehicle and HS-173 treatment. However, HS-173 treatment revealed differences in active myosin distribution at apical vertices (see arrowheads) and a more compact organization of the monolayer compared to vehicle control (**E**,**G**). 3D structures from confocal z-stacks, which were labeled for F-actin (white) and nuclei (DAPI, blue) were created to allow the better visualization of the compact organization of wounded epithelial follower cells with HS-173 and vehicle treatment (**H**,**I**). (**J**,**K**) Orthogonal views from confocal z-stacks of cultures labeled for F-actin (white) and nuclei (DAPI, blue) were created to reveal height differences in the wounded epithelial cells of HS-173 vs. DMSO-treated cultures. (**L**,**M**) 3D structures of orthogonal views were created from the same region in (**J**,**K**) to further highlight shape differences. Magnification bars = 20 μm (**B**–**G**,**J**,**K**) or 5 μm (**H**,**I**). (**B**–**G**) arrows show direction of cell migration.

**Figure 6:**
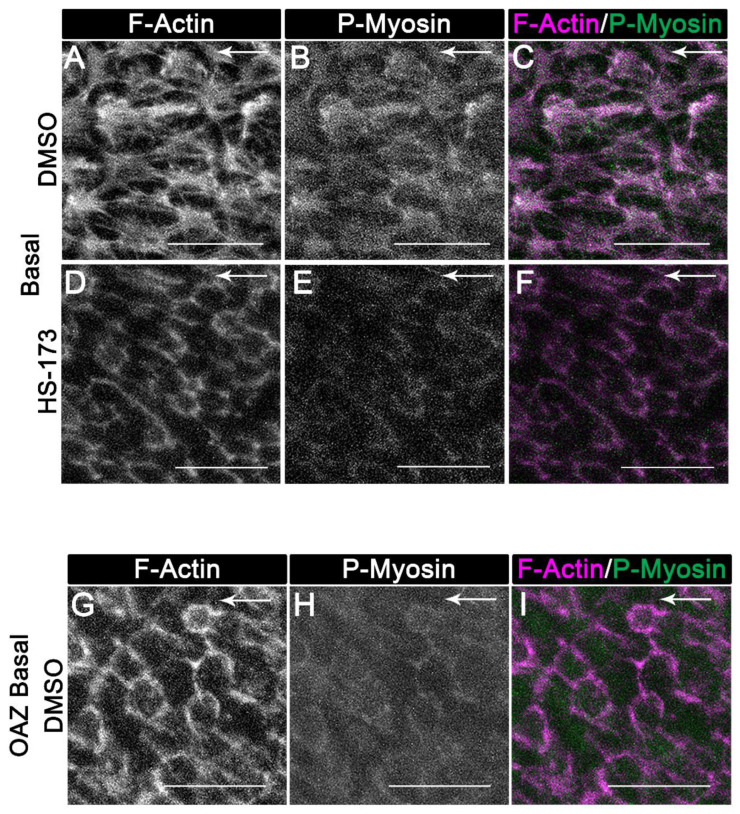
**Role for p110α in regulating reorganization of actomyosin along the basal surface of the lens epithelial follower cells for wound healing.** (**A**–**F**) Ex vivo post-cataract surgery explants were treated with vehicle control or HS-173 to inhibit p110α from Time 0 for 24 h (Day 1 post-injury). Cultures were labeled with phalloidin to identify filamentous actin (F-actin, white (**A**,**D**,**G**), purple (**C**,**F**,**I**)) and immunolabeled for active myosin II (P-myosin, white (**B**,**E**,**H**), green (**C**,**F**,**I**)). Confocal images along the basal surface of the wounded lens epithelium revealed that (**A**–**C**) normal basal actomyosin reorganization into stress fibers within wounded epithelial cells is blocked with (**D**–**F**) HS-173 treatment. (**G**–**I**) Confocal images along the basal surface of the wounded epithelial cell located in the OAZ are shown for comparison to HS-173 treated cells (**D**–**F**) revealing a similarity in basal actomyosin organization of HS-173 basal follower cells to nonmoving wounded epithelial cells in the OAZ. Magnification bars = 20 μm and arrows show direction of cell migration (**A**–**I**).

To examine the mechanism of how p110α promotes collective migration following wounding, we determined the specific effects of blocking p110α on the wounded lens epithelium, the follower cells in our culture model. Our previous studies show that actomyosin mechanical cues are critical for the organization of the wounded lens epithelial cells within the collectively migrating monolayer [7]. Active (phospho) myosin was found to localize to apical junctional complex that maintains cell–cell contact between wounded epithelial cells [7]. At the same time, the organization of actin stress fibers is induced along the basal cell compartment of the migrating epithelium where it is linked to cadherin junctions that are located in cryptic lamellipodia. This mechanical coupling is driven by active myosin to promote movement of the epithelium along the substrate [7]. Here, we investigated whether p110α signaling has a role in the regulation of apical and/or basal actomyosin organization in the wounded lens epithelial cells (modeled in Figure 5A). For these studies, ex vivo wounded cultures were treated with vehicle (DMSO) or the p110α inhibitor, HS-173 on day 0 through day 1 (Figure 5). The wounded explant cultures were immunolabeled for active myosin using an antibody that recognizes dually phosphorylated (Thr18/ser19) regulator light chain (RLC) of myosin II (Figure 5C,D,F,G; Figure 6B,C,E,F,H,I) and co-labeled for F-actin with fluorescent-conjugated phalloidin (Figure 5B,D,E,G,H–M; Figure 6A,C,D,F,G,I). Confocal z-stacks were acquired to determine changes in actomyosin organization and/or myosin activity along the apical (Figure 5B–G,J–M) and basal (Figure 6) domains of the wounded lens epithelial follower cells.

Confocal microscopy images focused along the apical surfaces of the migrating wounded epithelial cells revealed that exposure to the HS-173 inhibitor treatment decreased active (phospho)myosin at the apical vertices of the wounded lens epithelial cells (Figure 5C,F, arrowhead). Importantly, labeling of F-actin demonstrated that blocking p110α activation maintained the normal tightly packed columnar morphology of the lens epithelium (Figure 5E,K,M). Vehicle controls show the flattened migratory phenotype of the lens epithelium (Figure 5B,J,L) that was previously shown to characterize the collective migration of lens epithelial cells post-wounding [7]. The morphology of the lens epithelium in the presence of the HS-173 inhibitor also resembles the wounded epithelial cells at time 0 post-injury (Supplemental Figure S2A). 3D surface structures created from confocal z-stacks of cultures labeled for F-actin and nuclei highlight the compact organization of wounded epithelial follower cells with HS-173 treatment compared to vehicle alone (Figure 5H,I).

While inhibiting p110α signaling did not affect the distribution of apical F-actin, the wound-induced organization of basal actin structures was blocked (Figure 6A compared to Figure 6D). In vehicle controls, F-actin stress fibers were induced to form that co-localized with active (phospho) myosin (Figure 6A–C). These actomyosin filaments are expected to be linked to N-cadherin that localization to cryptic lamellipodia [7], and propel the wounded epithelial follower cells along the basal substrate. In the HS-173 treated wounded explants F-actin maintained a cortical distribution and at these cell–cell contacts F-actin was co-localized with active myosin (Figure 6D–F). Interestingly, the cortical distribution of actin along the basal surfaces of the wounded epithelial cells with HS-173 treatment parallels that of wounded epithelial cells located in the OAZ at time 0 post-injury ([7], Supplemental Figure S2B). Additionally, for comparison, confocal images of wounded epithelial cells from the OAZ of vehicle control were imaged along their basal surfaces at day 1 post-injury following labeling for F-actin and active myosin (Figure 6G–I). In contrast to wounded lens epithelial cells migrating across the cell-denuded basement membrane of the CMZ, F-actin and active myosin were distributed cortically in the wounded lens epithelial cells that remain associated with the OAZ (Figure 6G–I). These results show that lens epithelial follower cells treated with HS-173 share a similar cortical organization of actomyosin along their basal surfaces as the nonmotile, wounded lens epithelial cells in the OAZ of vehicle controls. These findings suggest that p110α signaling is needed to induce reorganization of actomyosin along the basal domains of the wounded epithelial cells for collective migration during wound healing.

### 3.4. p110α Is Required for Epithelial Cell Proliferation in Response to Wounding

**Figure 7:**
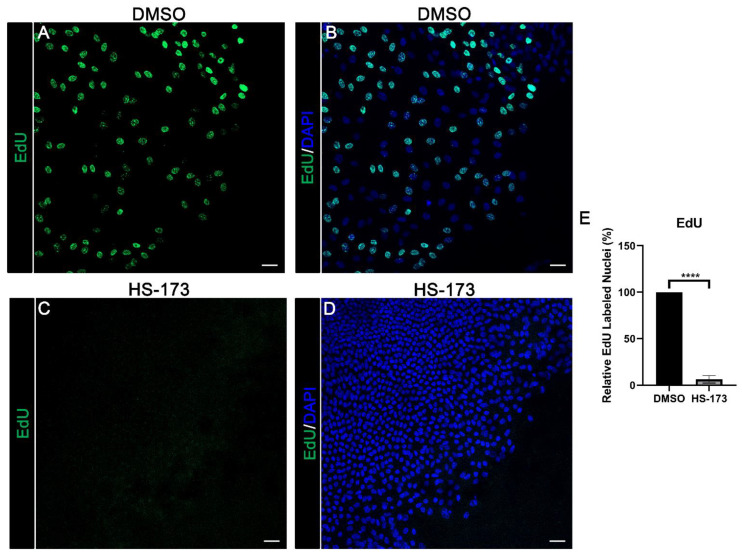
p110α is required for promoting cell proliferation in the central migratory zone during wound healing. (**A**–**E**) Time 0 ex vivo post-cataract surgery explants were treated with vehicle (DMSO) or HS-173 to inhibit p110α for 24 h (Day 1 post-injury) and labeled for 30min with EdU (green) (**A**,**C**) and counterstained with DAPI (blue) (**B**,**D**). (**E**) Graph depicts the change in the relative % of EdU positive cells within the CMZ region in vehicle compared to HS-173 treated cultures. Cell proliferation was blocked with HS-173 treatment. Vehicle was normalized to 100%. *p*-values were determined from unpaired *t*-test (**E**, **** *p* < 0.0001). Data is expressed as ± SEM from 3 independent experiments (**E**). Magnification bars = 20 μm (**A**–**D**).

In the lens, p110α function has previously been linked to regulating lens epithelial cell proliferation during lens development [33]. Since wound healing typically requires both collective migration and proliferation for wound closure, we next determined whether p110α may also regulate proliferation in response to wounding. Ex vivo post-cataract surgery cultures were treated with vehicle or HS-173 on day 0 and EdU labeled for 30 min on day 1 post-treatment. Cultures were counterstained with DAPI to identify nuclei and imaged at the wound edge of the CMZ. Treatment with HS-173 resulted in a major block in proliferation compared to vehicle control (Figure 7A–E). These findings show that p110α is critical to signal proliferation for re-epithelialization associated with wound closure in response to cataract surgery wounding.

## 4. Discussion

The PI3K p110α catalytic subunit has been linked to the development of fibrosis associated with hepatic stellate cells in liver fibrosis [34]. In a previous study from our lab of a post-cataract surgery model we showed that signaling through this PI3K isoform was associated with the emergence and expansion of myofibroblasts, the cellular culprits of lens fibrotic disease [31]. Here, we have focused on the mechanisms of collective migration of the epithelium to close the cataract surgery-induced wound. Using a wound healing model, our studies revealed that collective migration and proliferation for wound healing occur in a PI3K isoform-specific manner in response to cataract surgery injury. We found that PI3K p110α is an essential signaling cue to orchestrate proliferation and the distinct reorganization of polarized cytoskeletal activities of both resident immune leader cells and wounded lens epithelial cells for their collective movement in response to injury. Furthermore, we found that the PI3K/Akt signaling axis alone was insufficient to promote p110α induced collective migration, suggesting the involvement of other downstream effectors of PI3Kp110α signaling such as Rac, similar to the role of p110α in fibrosis [31]. For efficient movement it is necessary for polarized cells to extend protrusions persistently toward the direction of a chemotactic signal [15,35,36,37]. Our studies uncovered a key role for p110α in regulating the ability of leader cells to polarize, initiate, and maintain extended lamellipodia protrusions at the wound edge, critical to propel the directional migration of the connected group of epithelial cells forward toward the center of the wound. At this same time, p110α was required in wounded lens epithelial cells to signal reorganization of the actomyosin cytoskeleton into stress fibers along their basal domains. Here, actomyosin-rich stress fibers interconnect cryptic lamellipodia of each follower cell to mechanically couple the wounded epithelial cells to help drive the monolayer forward behind the leading edge for wound closure. Overall, our studies reveal p110α as a central coordinator of the wound healing response to cataract surgery injury.

Prior studies link PI3K, and PI3K p110α subunit to regulating cellular protrusion formation through impacting actin dynamics [13,38,39,40,41]. The results of the present investigation uncover a role for PI3K p110α in both initiating the formation of these protrusive processes as well as in the maintenance of these vimentin-rich lamellipodia extensions by resident immune cells that is critical for their persistent movement. In the cataract surgery wound healing model, leader cells extend vimentin-rich lamellipodia that are actin poor [26] and we previously showed that vimentin function is required for lamellipodial extension by leader cells at the wound edge and for proper wound closure [26]. Our new findings link the PI3K p110α isoform as an upstream regulator of vimentin intermediate filament dynamics of leader cells. How PI3K p110α regulates vimentin organization in leader cells is an intriguing question for future investigations. Prior studies indicate a role for PI3K in the regulation of vimentin organization. In a study with endothelial cells, PDGF was shown to induce reorganization of vimentin in a PI3K dependent manner [42]. In that study, inhibiting PI3K prevented the PDGF induced retraction of vimentin from the endothelial cell membrane [42]. In another study, PI3Kγ was shown to regulate leukocyte transendothelial cell migration through its ability to phosphorylate vimentin to induce vimentin disassembly [43]. In both these studies, PI3K has a role in signaling the disassembly of vimentin filaments. In contrast, our studies suggest that PI3K is necessary for organizing vimentin into polarized lamellipodial structures extended on the substrate by leader cells. The discrepancy in these findings suggests that PI3K regulates vimentin dynamics in a cell and context-dependent manner. Another possibility is that PI3K is necessary to create the polarizing signal at the membrane for vimentin filaments to be organized and extended at the leading edge in the direction of wound repair. In dictostyelium an amplified gradient of PIP3 is created at the leading edge to polarize the cell, creating a “cellular compass” for the cell to move in the direction of a chemoattractant gradient [13,16]. The chemokine SDF-1 is a possible upstream regulator of PI3K for chemokine-directed migration of the leader cells. SDF-1 can activate PI3K to induce accumulation of PIP3 within cells, such as T-cells [44,45] and we previously found that gene expression of SDF-1 and its receptor CXCR4 are upregulated 1hr post-cataract surgery injury [46]. Future studies are needed to better understand the molecular mechanism(s) by which PI3K p110α regulates polarized vimentin organization within leader cells for lamellipodial formation and directional movement during wound healing.

Coincident with the requirement for PI3K p110α induced changes in leader cells to promote lamellipodia protrusions we found that p110α is also critical for inducing changes in cell shape and cytoskeletal organization of the wounded epithelial cells that is linked with their transition from a stable epithelium to a migrating cell monolayer. There are several scenarios by which PI3K p110α injury induced signaling may regulate the wounded epithelial cells to undergo changes to a migratory phenotype. One involves the ability of PI3K p110α to signal spatiotemporal control over actomyosin stress fiber formation along the basal domains of the wounded epithelial cells through the activation of RhoGTPases that are linked to regulating actin cytoskeleton dynamics [22,47]. It is also possible that loss of polarized cytoskeletal function in leader cells to form persistent protrusions for directional migration by inhibition of PI3K p110α impacts signaling cues to the wounded follower cells necessary to establish front-to-rear polarity for collective migration. Our previous study showed that ablation of leader cell function slowed wound healing [30]. Whether leader cell function is critical to communicate changes to the wounded lens epithelial cells to coordinate their distinct polarized cytoskeletal activities for collective movement is not yet clear. Furthermore, we did not observe alterations in the cortical distribution of actin or myosin at apical cell–cell junctions when PI3K p110α function was inhibited. This does not preclude the possibility that dynamics of cadherin cell–cell junctions are altered which would contribute to the inability of the epithelial cells to undergo dynamic changes to undergo movement. In support of this possibility, PI3K p110α was shown to regulate endothelial cell–cell adherens junctions, which became strengthened when p110α was inhibited through a mechanism that involving cadherin tyrosine phosphorylation [48]. It is also possible that p110α induced proliferation is necessary for collective migration in response to cataract surgery wounding. An interesting study in Zebrafish kidney development showed a link between migration and proliferation [49]. In this study, collective migration induces cell stretch signals to promote PI3K dependent proliferation and blocking proliferation led to an inhibition of collective migration for kidney morphogenesis [49].

Overall, our studies reveal PI3K p110α as a critical coordinator of proliferation and polarized cytoskeletal dynamics within leader and follower cells to promote collective migration for proper wound healing in response to injury. While beyond the goals of the current study, it will be of future interest to elucidate whether PI3K p110α signals these processes through the same or distinct downstream effector(s). Lastly, our studies show that the post-cataract surgery model is a useful tool to exploit to better understand heterotypic leader and follower cell interactions for collective movement, which may provide insight into how this process leads to pathological outcomes such as cancer progression.

## Data Availability

Not Applicable.

## Supplementary Materials

The following supporting information can be downloaded at: https://www.mdpi.com/article/10.3390/cells11213515/s1, Figure S1: PI3K class I isoform expression in response to cataract surgery wounding; Figure S2: F-Actin organization within wounded lens epithelial cells at T0 post-injury.
