# Supplementary material for: PI3K Isoform-Specific Regulation of Leader and Follower Cell Function for Collective Migration and Proliferation in Response to Injury
Source: Cells. 2022 Nov 7;11(21):3515. doi: 10.3390/cells11213515 (PMC9658457; doi:10.3390/cells11213515)
Supplement: Supplementary file 1 [file cells-11-03515-s001.zip › cells-1982858-supplementary.pdf]

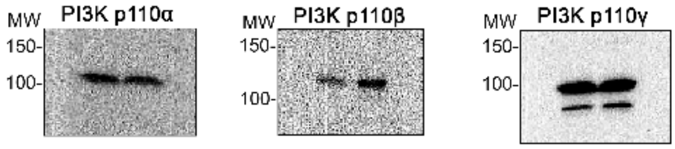

Supplemental Figure S1: PI3K class I isoform expression in response to cataract surgery wounding.
Western blot analysis shows that class I PI3K p110 isoforms, p110α, p110β and p110γ are all expressed in the lens in response to injury (Time 0).

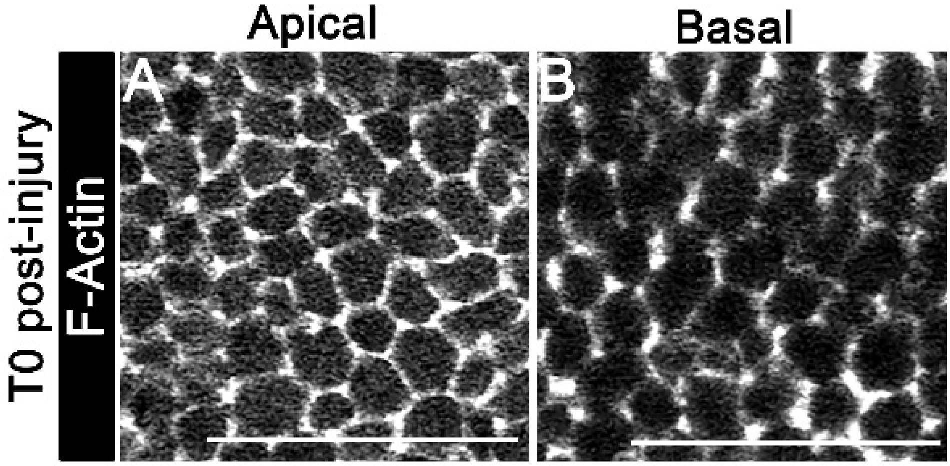

Supplemental Figure S2: F-Actin organization within wounded lens epithelial cells at T0 post-injury.
Cultures were labeled with phalloidin to detect F-actin (F-actin, white, A,B). Confocal images within the OAZ show the distribution of F-actin at the apical (A) and basal (B) aspects of the wounded epithelium. Magnification bar: 20μm (A, B).
